# Poisons, Their Effects and Detection

**Published:** 1886-01

**Authors:** 


POISONS, THEIR EFFECTS AND DETECTION. A manual for the use of analytical chemists and experts, with an introductory essay on the Growth of Modern Toxicology. By Alexander

Wynter Blyth, M. R. C. S., F. C. S., etc., Public Analyst for the County of Devon, and Medical Officer of Health and Public Analyst for St. Mangleton, with tables and illustrations. Volume II. New York: William Wood & Co., 56 and 58 Lafayette Place. 1885.

This number for July, sustains fully the favorable notice given previously of the first volume of this work, by one of the standard medical authors, whose productions constitute this excellent series of publicaticns.

